# An LC/MS/MS method for quantifying 25-hydroxyvitamin D_3_ in finger-prick plasma sample prepared by DEMECAL^®^ micro plasma separation device: toward mail-in assessment of vitamin D status

**DOI:** 10.1007/s00216-025-05939-4

**Published:** 2025-06-10

**Authors:** Kodai Maeda, Kyoka Sakamoto, Eiko Ito, Shinya Sugimoto, Shingo Tajima, Takahisa Sasahara, Shunji Kawamura, Kazuo Yaegashi, Tatsuya Higashi

**Affiliations:** 1 Faculty of Pharmaceutical Sciences, Tokyo University of Science, 6-3-1 Niijuku, Katsushika-ku, Tokyo 125-8585 Japan; 2 Leisure, Inc., 2-33-8 Nihombashi Ningyocho, Chuo-ku, Tokyo 103-0013 Japan; 3 Itabashi Medical Laboratory, EIL, Inc., 2-20-10, Azusawa, Itabashi-ku, Tokyo 174-0051 Japan

**Keywords:** Micro plasma separation, Finger-prick blood, Vitamin D status, LC/ESI–MS/MS, Derivatization

## Abstract

A mail-in assessment of vitamin D status will make it possible for the subjects to early detect any vitamin D insufficiency/deficiency at their homes, thus leading to a better management of their health. In this study, a liquid chromatography/electrospray ionization-tandem mass spectrometry (LC/ESI–MS/MS) method was developed and validated for quantifying 25-hydroxyvitamin D_3_ [25(OH)D_3_], which is the established marker of the vitamin D status, in the finger-prick blood plasma sample prepared by the DEMECAL^®^ device. By using the DEMECAL^®^ device, the subjects can collect their finger-prick blood at their homes and immediately separate the plasma from the blood by themselves. The DEMECAL^®^-based procedure had advantages over the venipuncture- and dried blood spot-based procedures because it is less invasive and a medical professional-free blood collection, and no need to be concerned with the heterogeneity of the analyte distribution in the sample. The developed LC/ESI–MS/MS method enabled the precise and accurate quantification of 25(OH)D_3_ and provided the lower limit of quantification of 0.10 ng/mL in the diluted plasma sample due to the derivatization with 4-(4-dimethylaminophenyl)-1,2,4-triazoline-3,5-dione. 25(OH)D_3_ was stable in the plasma sample prepared by the DEMECAL^®^ device at room temperature (23–27 °C) for 7 days (simulated condition in the mail) and at 4 °C for 30 days (simulated condition during storage at a laboratory). The measured 25(OH)D_3_ concentrations by the DEMECAL^®^-based procedure well agreed with those by the conventional venipuncture-based procedure. These results demonstrated that the derivatization-LC/ESI–MS/MS combined with the DEMECAL^®^-based sample preparation is a promising procedure for the mail-in assessment of the vitamin D status.

**Graphical Abstract:**

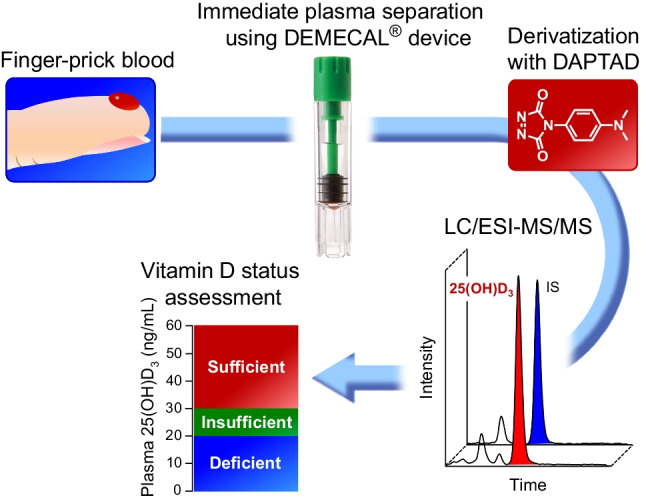

## Introduction

Vitamin D deficiency causes not only bone diseases as typified by rickets and osteoporosis, but also the increased risk of several non-bone diseases including diabetes [1], cardiovascular diseases [2], cancer [3], and depression [4]. The rising health consciousness has led to the increased demand for the assessment of the vitamin D status (sufficiency/deficiency). Although vitamin D actually consists of two different forms, i.e., vitamin D_2_ (D_2_) and vitamin D_3_ (D_3_), the latter is the predominant form in humans; the plasma/serum D_2_ and its metabolite concentrations are usually less than the quantifiable levels unless the subject takes D_2_-enriched foods, such as sun-dried mushrooms and D_2_ supplements [5–7]. D_3_ is not just derived from one’s diet but also endogenously synthesized, then hydroxylated in the liver to 25-hydroxyvitamin D_3_ [25(OH)D_3_], which is the circulating metabolite with a relatively long half-life (2–3 weeks [8]). 25(OH)D_3_ is further metabolized in the kidney to the active metabolite, 1,25-dihydroxyvitamin D_3_. It is widely accepted that the plasma/serum 25(OH)D_3_ concentration is the most useful index for the assessment of the vitamin D status [8–10], though several studies have demonstrated the values of the quantification of the plasma/serum 24,25-dihydroxyvitamin D_3_ as well as 25(OH)D_3_ [11–13], or analysis of the urinary D_3_ metabolites [14, 15] for this purpose.

For the assessment of the vitamin D status, the subject usually needs to have venous blood taken in a medical setting. If a mail-in service for the assessment of the vitamin D status is available, it becomes very convenient for the subjects because they do not need to go to a medical setting. For this service, a minimally invasive sample collection with no medical professionals is important. A self-collected finger-prick blood can be a sample fulfilling these needs. An excellent agreement was observed between the venous and capillary serum 25(OH)D_3_ concentrations [16, 17]. The dried blood spot (DBS) sample is one of the finger-prick blood samples being collected onto a specific paper. DBS sampling has been used to determine the circulating vitamin D metabolite levels [7, 18–22], but it is not without concerns; these include heterogeneity of the vitamin D metabolite distribution in the spot due to the varying collected blood volumes and individual hematocrit levels [19, 21]. The heterogeneity is particularly noticeable at the edges [23], which can be a major cause of inaccuracy in the DBS-based assays. Some studies also demonstrated that the 25(OH)D_3_ concentrations measured by the DBS-based methods were significantly lower than those in the venipuncture samples (plasma/serum) [7, 20]. Recently, the Mitra^®^ device (Neoteryx, CA, USA) based on the volumetric absorptive microsampling (VAMS^®^) technology was applied to measure the circulating 25(OH)D levels [6]. The VAMS^®^ is another approach to obtain a dried blood sample for quantitative analysis and enables collecting a fixed volume (typically 20 μL) of blood without perturbation by hematocrit. It was reported that the variation in the collected blood volume was 5% over a hematocrit range of 20–70% by the VAMS^®^ [23], while that of the DBS sub-punches was 30% over the same hematocrit range [24]. In the assays using the VAMS^®^, whole samples are used for the quantitative analysis. Thus, the VAMS^®^ overcomes the homogeneity issue associated with a conventional DBS sampling. However, the tip dipped in the blood is required to be dried at room temperature for at least 2 h along with preventing the tip from touching others; this drying process is time-consuming and might be a major limitation of the VAMS^®^-based Mitra^®^ device.

In this study, another sampling device, DEMECAL^®^, developed by Leisure (Tokyo, Japan) was used. By using this device, the subjects (medically unskilled persons) can collect their finger-prick blood and directly separate the plasma from the blood by a simple operation by themselves. The plasma volume taken for the analysis can be correctly determined by the aid of a marker compound to calculate the dilution factor even if the collected blood volume and hematocrit are varied. Because the liquid sample (diluted plasma) is used in the DEMECAL^®^-based assay, the heterogeneity of the analyte distribution is excluded. The diluted plasma samples prepared by the DEMECAL^®^ device simplify workflow in the laboratory; an extraction process from the dried blood samples (DBS and VAMS^®^ samples) is not necessary. Thus, the DEMECAL^®^-based procedure offers some advantages over the DBS- and Mitra^®^-based procedures. The procedure of the DEMECAL^®^ device is described in the “Experimental” section.

The DEMECAL^®^ device has been used for the mail-in check of lifestyle-related diseases, and test items include AST, ALT, γ-GT, total protein, and albumin for liver function; BUN and creatinine for kidney function; glucose and HbA1c for sugar metabolism; total-, HDL-, and LDL-cholesterol, and triglyceride for lipid metabolism; and uric acid for the gout test [25]. However, this mail-in service is not currently applied to the assessment of vitamin D status due to the lack of a validated method for quantifying 25(OH)D_3_ in the finger-prick plasma sample prepared by the DEMECAL^®^ device. If the DEMECAL^®^-based assessment of vitamin D status is available, the subjects can better perceive their own health at their homes. To achieve this, a method that enables the quantification of 25(OH)D_3_ in a small volume (≈ 10 μL) of the finger-prick plasma sample diluted with the storage buffer (corresponding to ca. 1 μL of undiluted plasma) is required. We have developed 4-(4-dimethylaminophenyl)-1,2,4-triazoline-3,5-dione (DAPTAD) as the derivatization reagent to increase the detectability and specificity of the vitamin D compounds for their liquid chromatography/electrospray ionization-tandem mass spectrometry (LC/ESI–MS/MS) quantification [26]. It has been proved that DAPTAD works well for the trace and accurate quantification of vitamin D metabolites in small volume blood-derived samples [11, 20, 27, 28].

Based on this background information, our final goal was to develop the DEMECAL^®^-based mail-in procedure for the assessment of the vitamin D status. For this purpose, in this study, a method was developed and validated for quantifying the trace level of 25(OH)D_3_ in the finger-prick plasma sample prepared by the DEMECAL^®^ device by LC/ESI–MS/MS combined with the DAPTAD derivatization.

## Experimental

### Chemicals and reagents

25(OH)D_3_ and [6,19,19-^2^H_3_]-25(OH)D_3_ (internal standard (IS)) were purchased from the FUJIFILM Wako Pure Chemical Corporation (Osaka, Japan) and IsoSciences (King of Prussia, PA, USA), respectively, and dissolved in ethanol to prepare the 1.0 μg/mL stock solutions. The 25(OH)D_3_ working solutions of 100, 200, 500, 1000, 2000, 5000, 10,000, 20,000, and 50,000 pg/mL were prepared by subsequential dilutions of the stock solution with ethanol. The working solutions of IS in ethanol (1.0 or 5.0 ng/mL) were also prepared. 3-Epi-25(OH)D_3_ was purchased from the Cayman Chemical Company (Ann Arbor, MI, USA). DAPTAD was synthesized in our laboratory by a known method [26]. Methanol and ammonium formate used for the mobile phase were of LC/MS grade (FUJIFILM Wako Pure Chemical Corporation). Ultrapure water was prepared by a Puric-α system (Organo, Tokyo). All other reagents and solvents used for sample pretreatment and derivatization were of analytical grade from the Tokyo Chemical Industry (Tokyo, Japan).

### DEMECAL^®^ device

The DEMECAL^®^ device was donated from Leisure and contains (1) a lancet to prick the finger, (2) an absorbent polymer tip (sponge) attached to a stick-type mounting to absorb ca. 65 μL of the blood, which generally contains ca. 30–40 μL of plasma, (3) a cylinder-shaped container containing a storage buffer (HEPES buffer (pH 7.4), 280 μL), and (4) a piston-shaped plasma separator (blood cell/plasma separation membrane). An antiseptic cotton and bandage are also included (see https://www.leisure.co.jp/products# for more information).

**Fig. 1:**
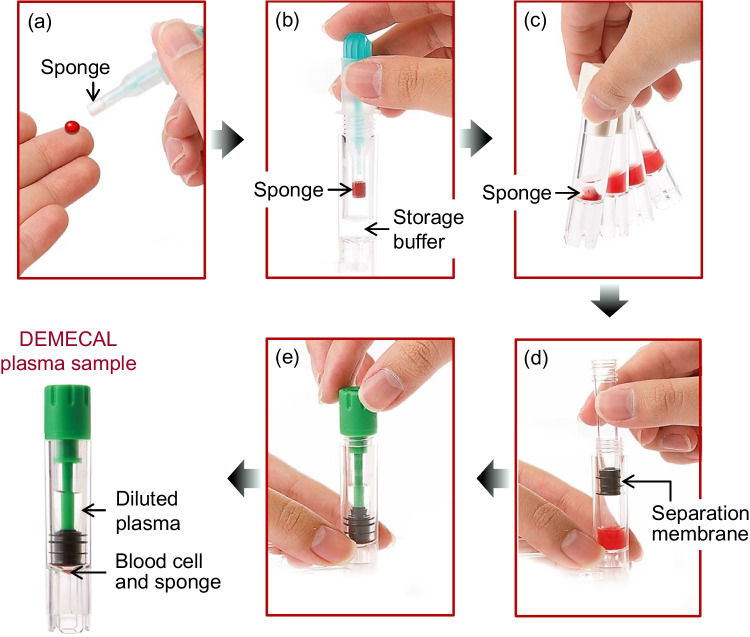
Outline drawing of preparing DEMECAL plasma sample

The procedure using the DEMECAL^®^ device is as follows. The sponge is dipped in a drop of capillary blood from the puncture until the tip is entirely red (Fig. 1a). The sponge is placed in the container containing the storage buffer by pushing the button (Fig. 1b). The container is shaken until the storage buffer turns uniformly red (Fig. 1c). The piston-shaped plasma separator is set on the container and slowly slid down to separate the plasma (Fig. 1d); approximately 8–10 times diluted plasma is isolated above the separation membrane, and the sponge and blood cells remain underneath the membrane. The container of the plasma sample is covered with a containment cap (Fig. 1e), then mailed to a certified laboratory. The storage buffer contains a marker compound (undisclosed due to confidential corporate information) to calculate the dilution factor of the plasma; by measuring the concentration of this marker compound, the plasma volume taken for the analysis can be correctly determined if the collected blood volume and hematocrit are varied [25].

### Blood samples

Finger-prick blood plasma samples were prepared by the DEMECAL^®^ device according to the manufacturer’s instructions and stored in the container with the storage buffer at room temperature unless otherwise noted; these samples were designated as the “DEMECAL plasma samples” in this study. In most cases, the DEMECAL plasma samples were analyzed on the same or next day that the sample collection was done. The blood samples of some subjects were also collected from their median cubital veins into an INSEPACK^®^ II-ST tube (Sekisui Medical, Tokyo), left for 30 min at room temperature, then centrifuged (2000 × *g*, 15 min). The separated sera were stored at − 30 °C until used; these samples were designated as the “venipuncture serum samples”. Apparently healthy subjects of both sexes (19–62 years old) participated in this study and donated their blood samples with the full understanding of the purpose of this study and their written informed consents. The experimental procedures were approved by the Institutional Review Board of Tokyo University of Science (No. 24027; Tokyo) and Itabashi Chuo Medical Center (No. 241022 A; Tokyo).

### Pretreatment of DEMECAL plasma sample

The DEMECAL plasma sample (10 μL) was added to the mixture of acetonitrile (180 μL) and the IS working solution (10 pg in ethanol (10 μL)), then vortex-mixed for 30 s for deproteinization. After the centrifugation (1000 × *g*, 10 min), the supernatant (150 μL) was transferred to another tube. After evaporation of the solvent under N_2_ at 40 °C, the residue was dissolved in the DAPTAD solution (10 μg in ethyl acetate (50 μL)), then stored at room temperature for 1 h. Ethanol (20 μL) was added to the mixture to terminate the reaction, and then the solvent was evaporated under N_2_ at 40 °C. The resulting derivatives were dissolved in the mobile phase (50 μL), 10 μL of which was injected into the LC/ESI–MS/MS.

### LC/ESI–MS/MS

A Shimadzu LCMS-8045 triple quadrupole mass spectrometer connected with a Shimadzu LC-40D XS chromatograph (Kyoto, Japan) was used. Methanol-10 mM ammonium formate (10:3, v/v, isocratic mode) was used as the mobile phase and run through the Ascentis Express 90Å C18 (2.0 µm, 100 × 2.1 mm i.d.; Sigma-Aldrich Japan, Tokyo) column at the rate of 0.3 mL/min and a temperature of 40 °C. The ESI–MS/MS conditions (positive-ion mode) were as follows: interface voltage, 4.0 kV; detector voltage, 1.82 kV; Q1 pre-rod bias voltage, − 30 V; Q3 pre-rod bias voltage, − 24 V; collision energy, − 25 V; nebulizer gas (N_2_) flow rate, 3 L/min; drying gas (N_2_) flow rate, 5 L/min; desolvation line temperature, 250 °C; heat block temperature, 400 °C; and collision gas (Ar), 230 kPa. The selected reaction monitoring (SRM) transitions (precursor and product ions) were *m*/*z* 619.5 → 341.3 and 622.5 → 344.3 for the derivatized 25(OH)D_3_ and IS, respectively. LabSolutions software (version 5.118, Shimadzu) was used for the system control and data processing.

### Calibration curve

The FFP-LR Nisseki frozen plasma (0.4 mL, the Japan Red Cross Service, Tokyo) was diluted with the storage buffer (2.8 mL), stirred with activated charcoal (0.32 g; Norit^®^, Nacalai Tesque, Kyoto) for 15 h, and then centrifuged (2000 × *g*, 10 min). The resulting supernatant, in which 25(OH)D_3_ was not detected, was used as the surrogate matrix for constructing the calibration curves. The surrogate matrix (10 μL) was added to the mixture of acetonitrile (180 μL) containing 25(OH)D_3_ (1.0, 2.0, 5.0, 10, 20, 50, or 100 pg) and the IS working solution (10 pg in ethanol (10 μL)); these corresponded to the calibration samples with the 25(OH)D_3_ concentration of 0.10, 0.20, 0.50, 1.0, 2.0, 5.0, or 10 ng/mL and the IS concentration of 1.0 ng/mL. The resulting sample was pretreated and derivatized as previously described. The peak area ratios (derivatized 25(OH)D_3_/IS; *y*) were plotted versus the 25(OH)D_3_ concentrations (ng/mL; *x*) to draw the calibration curve. The linear regression was fitted with a weighting factor of 1/*x*.

### Precision and accuracy

Two DEMECAL plasma samples were prepared from the second and third fingers of each subject, then mixed in a tube (total ca. 350 μL of the DEMECAL plasma sample). The mixed samples were stored in the DEMECAL^®^ containers on halves at 4 °C. Among the samples from several subjects, we found the samples with very low (#1), low (#2 and 5), medium (#3 and 6), and high (#4 and 7) concentrations of 25(OH)D_3_, which were used to assess the assay precision and accuracy. As these samples contained different levels of 25(OH)D_3_ (0.49–4.39 ng/mL in the DEMECAL plasma sample) as described in Tables 1 and 2, the assay precision and accuracy at the various concentrations could be tested. Intra-assay precision and accuracy were assessed by five repetitive measurements of these samples on 1 day. Subsequently, inter-assay precision and accuracy were assessed by duplicate measurements of the same samples (or the same samples spiked with 25(OH)D_3_) per day over five consecutive days. The assay precision and accuracy were evaluated based on the relative standard deviations (RSDs, %) of the measured concentrations and analytical recovery rates (%), respectively. The analytical recovery rates (%) were determined as follows. The DEMECAL plasma sample (10 μL) was added to the mixture of acetonitrile (180 μL) containing 25(OH)D_3_ (0 (non-spiked sample), 12.5, 25.0, or 50.0 pg (spiked sample); corresponding to spiked concentration of 0, 1.25, 2.50, or 5.00 ng/mL) and the IS working solution (10 pg in ethanol (10 μL)), pretreated, then derivatized. The resulting non-spiked and spiked samples were subjected to the LC/ESI–MS/MS. Analytical recovery rate (%) = [measured concentration of spiked sample/(measured concentration of non-spiked sample + spiked concentration) × 100 (%)].

**Table 1:** Assay precision

Sample	Intra-assay		Inter-assay	
	Measured (ng/mL)^a^	RSD (%)	Measured (ng/mL)^a^	RSD (%)
#1 (very low concentration)	0.49 ± 0.01	2.0	0.49 ± 0.01	2.0
#2 (low concentration)	1.62 ± 0.05	3.1	1.59 ± 0.06	3.8
#3 (medium concentration)	2.62 ± 0.03	1.1	2.59 ± 0.05	1.9
#4 (high concentration)	4.07 ± 0.13	3.2	4.01 ± 0.06	1.5

^a^Mean ± SD (*n* = 5)

**Table 2:** Assay accuracy

Sample	Spiked (ng/mL)	Intra-assay accuracy (%)^a^	Inter-assay accuracy (%)^a^
#5^b^	1.25	98.1 ± 4.4	98.9 ± 1.4
	2.50	97.5 ± 2.8	98.1 ± 1.9
#6^b^	2.50	96.2 ± 2.2	97.3 ± 2.9
	5.00	100.4 ± 2.9	96.5 ± 3.5
#7^b^	2.50	95.7 ± 2.0	99.1 ± 3.6
	5.00	95.4 ± 1.3	96.8 ± 1.5

^a^Mean ± SD (*n* = 5)

^b^The intrinsic 25(OH)D_3_ concentrations of samples #5, 6, and 7 were 1.04, 2.06, and 4.39 ng/mL, respectively

### Matrix effect

The peak areas (absolute values) of the DAPTAD-derivatized 25(OH)D_3_ and IS in the matrix samples were divided by those in the standard samples to determine the matrix effect, which was expressed as a percentage.

Standard sample: a mixture of 25(OH)D_3_ and IS (400 pg each) was derivatized and dissolved in the mobile phase (1.0 mL, *n* = 5). Matrix sample: the DEMECAL plasma sample was deproteinized, and then the supernatant was evaporated to dryness. To the obtained residue, one-twentieth the quantity of the above standard sample (50 μL) was added to prepare the matrix sample (*n* = 5).

### Stability

The stability of 25(OH)D_3_ in the DEMECAL plasma sample was evaluated by comparing the measured concentrations before and after storage. Five DEMECAL plasma samples collected from five different subjects were exposed to the following two different conditions: (1) at room temperature (23–27 °C) for 7 days, which simulated the condition in the mail and (2) at 4 °C (in refrigerator) for 1 month, which simulated the condition during the storage at a laboratory. The processed samples from five different subjects were also kept in the autosampler at 20 °C for 24 h. The DEMECAL plasma sample is usually not cryopreserved (the container is unfit for frozen storage); therefore, the freeze–thaw stability was not evaluated in this study.

### Quantification of 25(OH)D_3_ in venipuncture serum samples

Venous blood plasma/serum is currently the reference sample for assessment of the vitamin D status (measurement of 25(OH)D_3_). To validate whether a DEMECAL plasma sample could be an alternative to the venipuncture serum sample, the measured concentrations of both samples collected from the same subjects on the same day were compared. The concentrations of 25(OH)D_3_ in the venipuncture serum samples were determined by a previously reported method [29] with a slight modification. Briefly, the serum (5.0 μL) was deproteinized with the mixture of acetonitrile (85 μL) and the IS working solution (50 pg in ethanol (10 μL)), the supernatant was evaporated to dryness. The residue was derivatized with DAPTAD. The LC/ESI–MS/MS conditions were the same as those for the DEMECAL plasma sample. The correlation between the DEMECAL plasma and venipuncture serum 25(OH)D_3_ concentrations was analyzed by Spearman’s rank correlation coefficient. *p* < 0.05 was considered statistically significant.

## Results and discussion

### Pretreatment and LC/ESI–MS/MS conditions for quantifying 25(OH)D_3_ in the DEMECAL plasma sample

**Fig. 2:**
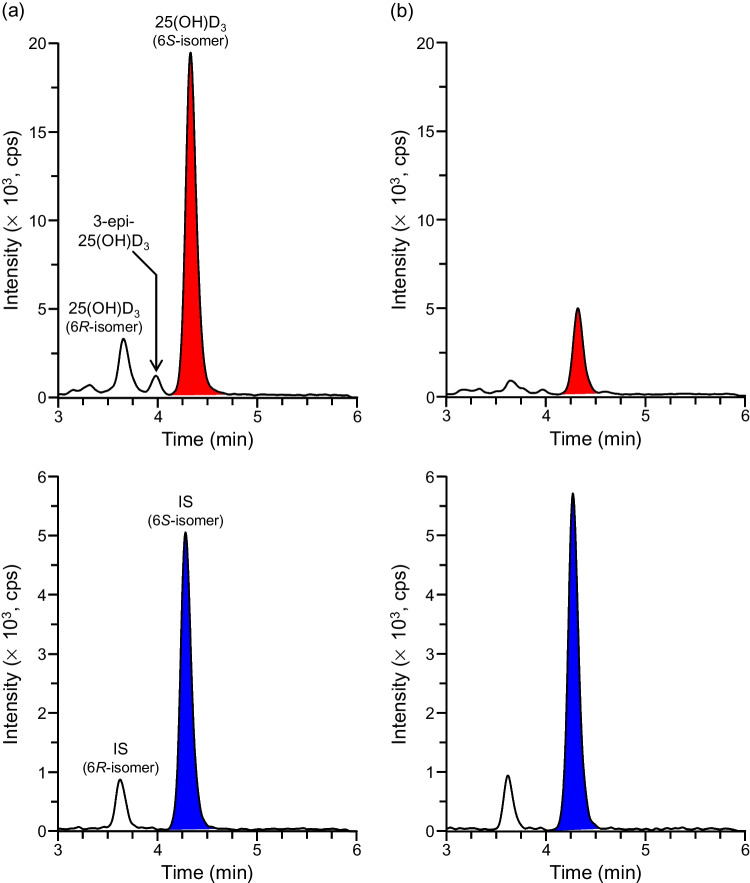
SRM chromatograms of DAPTAD-derivatized 25(OH)D_3_ and IS in DEMECAL plasma samples collected from **a** a subject with a sufficient vitamin D level and **b** a subject diagnosed with a vitamin D deficiency. The measured plasma concentrations were **a** 32.0 and **b** 8.1 ng/mL, respectively

As the DEMECAL plasma sample was the plasma diluted approximately eightfold–tenfold with the storage buffer, an aliquot (10 μL) for one analysis contained approximately 1 μL of undiluted plasma. This sample was deproteinized with acetonitrile, then derivatized with DAPTAD to impart the high proton-affinity (enhanced ESI efficiency) and specific fragmentation behavior (C6-7 bond cleavage) to 25(OH)D_3_ [26]. The derivatization reaction was carried out as previously reported because the derivatives were reproducibly and quantitatively produced under the reported conditions even for pg amounts of the D_3_ metabolites [14, 26]. The isomeric derivatives, i.e., 6*S*- (major isomer, retention time (*t*_R_) 4.3 min) and 6*R*- (minor isomer, *t*_R_ 3.6 min) isomers were formed under these conditions because DAPTAD attacked the *s*-*cis*-diene of 25(OH)D_3_ from the α- and β-sides of the vitamin D skeleton [26]. We verified that the peak area ratio of 6*R*/6*S*-isomers was constant (*R*:*S* = 1:6.2–1:6.5) regardless of the amount of 25(OH)D_3_ in the range of 1.0–100 pg/tube. The peaks corresponding to the 6*S*- (major) isomers of the derivatized 25(OH)D_3_ and IS (*t*_R_ 4.3 min) clearly appeared in their SRM chromatograms without interference from coexisting substances for all the samples analyzed in this study (Fig. 2). On the other hand, as shown in Fig. 2b, the 6*R*- (minor) isomer peak was detected with some interference from coexisting substances, which led to a poor peak shape, in a sample with low 25(OH)D_3_ concentration. Based on these results, it might be inadvisable to use a sum of major and minor peak areas for the quantitative analysis; only the major isomer peaks of the derivatized 25(OH)D_3_ and IS were used in the subsequent experiments. The Ascentis Express 90Å C18 column, which was packed with superficially porous particles (2.0 μm particle size and 1.2 μm core size), provided the sharper and more symmetrical peaks compared to the columns packed with fully porous octadecyl silica (ODS) materials, such as YMC-Ultra HT Pro C18 (2.0 µm, 100 × 2.0 mm i.d.) (YMC, Kyoto, Japan). An isocratic elution with the mobile phase of methanol-10 mM ammonium formate (10:3, v/v) was employed from the aspect of simplicity of the LC conditions and relatively short analysis time (6 min/sample). Ammonium formate facilitated the ionization of the DAPTAD derivative due to its acidic property. Under the stated LC conditions, the derivatized 25(OH)D_3_ could be satisfactorily separated from the interfering epimer, 3-epi-25(OH)D_3_ (*t*_R_ 4.0 min; Fig. 2) [22, 30]; the resolution value calculated from the half widths was 1.86. No matrix effects were observed for the derivatized 25(OH)D_3_ (100.7 ± 2.6%, mean ± standard deviation (SD), *n* = 5) and IS (100.9 ± 1.7%).

### Calibration curve

The regression formula was *y* = (1.00304 ± 0.00690)*x* + (0.00140 ± 0.00554) (mean values ± SDs were given for the slope and *y*-intercept of five curves), which demonstrated that the curves were reproducibly constructed (RSDs of the slope, 0.69%) and had a negligibly small *y*-intercept. A good linearity was also obtained in the range of 0.10–10 ng/mL as the determination constants (*r*^2^) were ≥ 0.999. The lower limit of quantification (LLOQ) was determined to be the same as the lowest calibration point (0.10 ng/mL) because the RSD of the back-calculated values (0.099 ± 0.005 ng/mL, mean ± SD, *n* = 5) was 5.1% and relative errors ranged from − 8.0 to 4.0%. The peak intensity at this concentration was at least 15 times greater than the noise intensity.

### Precision and accuracy

The RSDs of the intra- and inter-assay measurements were ≤ 3.2% and ≤ 3.8%, respectively, when four samples with different 25(OH)D_3_ concentrations were analyzed (Table 1). Both the intra- and inter-assay accuracies (analytical recovery rates) of all the tested samples were very close to 100% (Table 2). Thus, a good assay precision and accuracy were demonstrated.

### Stability

To achieve the DEMECAL^®^-based mail-in assessment of the vitamin D status, 25(OH)D_3_ in the DEMECAL plasma sample is required to be stable in the mail at ambient temperature. In Japan, the sample can be delivered to the laboratory by parcel post within a few days. The sample is then refrigerated at the laboratory until analyzed (for up to 1 week). Based on such a situation, the stability of 25(OH)D_3_ in the DEMECAL plasma sample at room temperature (23–27 °C) for 7 days and at 4 °C for 30 days was tested. As is obvious from Table 3, 25(OH)D_3_ was stable under these conditions (the measured 25(OH)D_3_ concentrations in the samples used in the stability tests ranged from 0.42 to 3.60 ng/mL). These results indicated that the preanalytical sample management does not become a bottleneck for the DEMECAL^®^-based mail-in assessment of the vitamin D status. Furthermore, the processed sample dissolved in the mobile phase was also stable in the autosampler (20 °C) for at least 24 h.

**Table 3:** Stability

Room temperature^a^	4 °C^b^	In autosampler^c^
100.6 ± 2.6	101.0 ± 2.7	102.6 ± 2.4

^a–c^Results are shown in % (mean ± SD, five different samples)

^a^Stability at room temperature (23–27 °C) for 7 days

^b^Stability at 4 °C for 30 days

^c^Stability of the pretreated and derivatized sample dissolved in the mobile phase at 20 °C for 24 h

### 25(OH)D_3_ concentrations determined by DEMECAL^®^-based procedure and correlation with those by the venipuncture-based procedure

The plasma 25(OH)D_3_ concentrations of apparently healthy Japanese adults determined by the DEMECAL^®^-based procedure were 18.1 ± 7.3 ng/mL (mean ± SD, range 7.3–38.0 ng/mL, *n* = 35). These concentrations were consistent with the reported reference interval of the Japanese adults, which was established using venipuncture (serum) samples: 15.5 ± 6.0 ng/mL (95% confidence interval 6–29 ng/mL,* n* = 5518) [5] and 20.0 ± 7.4 ng/mL (*n* = 9084) [31]. It has been reported that a great portion of Japanese people is diagnosed with vitamin D insufficiency (< 30 ng/mL of 25(OH)D_3_) or deficiency (< 20 ng/mL) [5, 31]. A trend that the 25(OH)D_3_ concentrations are lower in women (16.6 ± 5.8 ng/mL, mean ± SD, *n* = 14, 23.9 ± 9.6 years old) than in men (19.0 ± 8.1 ng/mL, *n* = 21, 33.1 ± 14.4 years old) was observed in this study as with the results in previous studies [5, 31], although a significant difference was not detected (*p* = 0.38, Mann–Whitney *U* test).

**Fig. 3:**
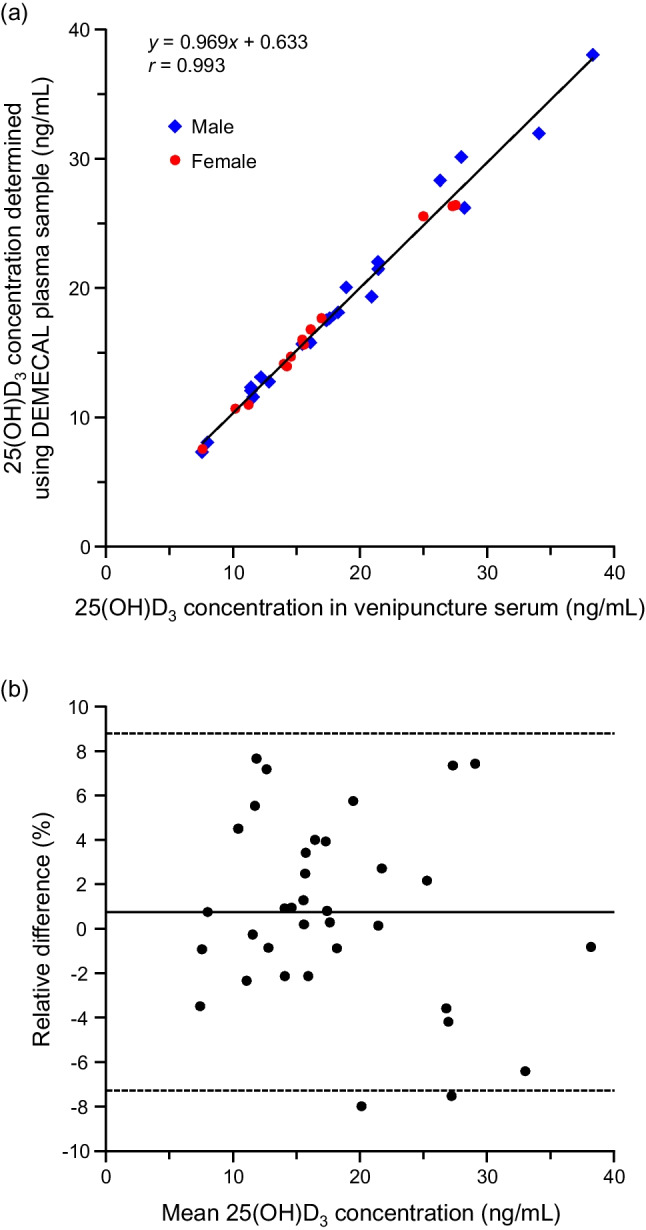
**a** Scatter plot to compare the measured 25(OH)D_3_ concentrations by the DEMECAL^®^-based procedure and the conventional venipuncture-based procedure (male, *n* = 21 and female, *n* = 14). **b** Bland–Altman plot of the mean 25(OH)D_3_ concentration and the relative difference between the DEMECAL^®^- and venipuncture-based procedures. The relative difference was calculated as follows: [(25(OH)D_3_ concentration by DEMECAL^®^-based procedure) − (25(OH)D_3_ concentration by venipuncture-based procedure)]/mean 25(OH)D_3_ concentration × 100 (%). The dashed lines represent the 95% limits of agreement

The vitamin D status has been conventionally assessed by the venous plasma/serum 25(OH)D_3_ levels [8–10]. In order to put the DEMECAL^®^-based procedure into practical use for the mail-in assessment of the vitamin D status, the correlation was assessed between the 25(OH)D_3_ concentrations determined using the DEMECAL plasma samples and those using the venipuncture serum samples collected from the same subjects on the same day (*n* = 35). This result was displayed as the scatter plot in Fig. 3a. A strong positive correlation (*p* < 0.001, Spearman’s rank correlation coefficient) was observed in the 25(OH)D_3_ concentrations between the DEMECAL and venipuncture samples; the linear regression line was (DEMECAL, *y*) = 0.969 × (venipuncture, *x*) + 0.633 with a correlation coefficient (*r*) of 0.993. The Bland–Altman plot showed that the difference in the 25(OH)D_3_ concentrations between the DEMECAL and venipuncture samples was negligibly small; the mean relative difference was 0.74% (range from − 8.0 to 7.7%) (Fig. 3b). These results demonstrated that the DEMECAL^®^-based procedure can be upward compatible with the conventional venipuncture-based procedure when it comes to the less invasive and medical professional-free sample collection. Furthermore, as mentioned in the “Introduction”, the 25(OH)D_3_ concentrations measured using the DBS samples were sometimes significantly lower than those using the venipuncture samples (plasma/serum) [7, 20], whereas it was shown in a recent study that the measured concentrations using the DBS and venipuncture samples well agreed when the collected blood volume and the punch-out part (central part of spot) were regulated in the DBS-based assay [18]. These data are also of help as evidence that the DEMECAL^®^-based procedure overcomes the issues related to the blood volume variation and hematocrit bias associated with conventional DBS-based assays.

## Conclusion

We developed and validated an LC/ESI-MS/MS method for quantifying 25(OH)D_3_ in the finger-prick plasma sample prepared using the DEMECAL^®^ device. The DEMECAL^®^ device enabled the subjects to easily collect their finger-prick blood at their homes and prepare the plasma directly after the blood collection by themselves. As the liquid sample (diluted plasma) was obtained by the DEMECAL^®^ device, the issue of the heterogeneity of the analyte distribution in the sample, which is associated with conventional DBS-based assays, was excluded. The DAPTAD derivatization followed by LC/ESI-MS/MS enabled the precise and accurate quantification of 25(OH)D_3_ and provided the LLOQ of 0.10 ng/mL in the diluted plasma sample, which was sensitive enough in order to easily detect the vitamin D deficiency. 25(OH)D_3_ was stable in the DEMECAL^®^ plasma sample under the simulated conditions in the mail and during the storage in a laboratory. The measured 25(OH)D_3_ concentrations by the DEMECAL^®^-based procedure well agreed with those by the conventional venipuncture-based procedure; the DEMECAL^®^-based procedure was proven to serve as a less invasive and medical professional-free alternative to the venipuncture-based procedure. This LC/ESI-MS/MS assay combined with the DEMECAL^®^ device would promise to achieve a mail-in assessment of the vitamin D status, by which the subjects can detect any vitamin D insufficiency/deficiency earlier at their homes and have a better management of their health.

## Data Availability

Data will be made available upon request.

## Declarations

### Source of biological material

The DEMECAL plasma samples were collected by the subjects themselves. The venipuncture serum samples were collected by a physician at the Itabashi Chuo Medical Center. Pooled human plasma FFP-LR (Nisseki frozen plasma) was purchased from the Japan Red Cross Service.
